# Injury Characteristics, Outcomes, and Health Care Services Use Associated With Nonfatal Injuries Sustained in Mass Shootings in the US, 2012-2019

**DOI:** 10.1001/jamanetworkopen.2022.13737

**Published:** 2022-05-27

**Authors:** Matthew P. Czaja, Chadd K. Kraus, Su Phyo, Patrick Olivieri, Dalier R. Mederos, Ivan Puente, Salman Mohammed, Ross P. Berkeley, David Slattery, Thomas H. Gildea, Claire Hardman, Brandi Palmer, Melissa L. Whitmill, Una Aluyen, Jeffery M. Pinnow, Amanda Young, Carly D. Eastin, Nurani M. Kester, Kaitlyn R. Works, Andrew N. Pfeffer, Aleksander W. Keller, Adam Tobias, Benjamin Li, Brian Yorkgitis, Soheil Saadat, Mark I. Langdorf

**Affiliations:** 1 Ponce Health Sciences University School of Medicine, Ponce, Puerto Rico; 2 Geisinger Emergency Medicine, Danville, Pennsylvania; 3 Touro University Nevada College of Osteopathic Medicine, Henderson; 4 Valley Health Emergency Medicine, Las Vegas, Nevada; 5 Division of Trauma and Critical Care Services, Broward Health Medical Center, Fort Lauderdale, Florida; 6 Department of Emergency Medicine, University of Nevada, Las Vegas Kirk Kerkorian School of Medicine, Las Vegas; 7 Department of Emergency Medicine, St Louise Regional Hospital, Gilroy, California; 8 Department of Emergency Medicine, Santa Clara Valley Medical Center, San Jose, California; 9 Department of Surgery, Wright State University Boonshoft School of Medicine, Dayton, Ohio; 10 Trauma Research Program, Kettering Medical Center, Kettering, Ohio; 11 Division of Acute Care Surgery, Critical Care, and Trauma, Department of Surgery, Kettering Medical Center, Kettering, Ohio; 12 Department of Emergency Medicine, Texas Tech University Health Sciences Center School of Medicine, Odessa; 13 Department of Emergency Medicine, Medical Center Hospital, Odessa, Texas; 14 Department of Emergency Medicine, University of Arkansas for Medical Sciences, Little Rock; 15 Department of Emergency Medicine, University of Texas Health Science Center at San Antonio; 16 Department of Emergency Medicine, Vanderbilt University Medical Center, Nashville, Tennessee; 17 Department of Emergency Medicine, University of Pittsburgh Medical Center, Pittsburgh, Pennsylvania; 18 Department of Emergency Medicine, Denver Health, Denver, Colorado; 19 Division of Acute Care Surgery, Department of Surgery, University of Florida College of Medicine, Jacksonville; 20 Department of Emergency Medicine, School of Medicine, University of California, Irvine

## Abstract

**Importance:**

Civilian public mass shootings (CPMSs) in the US result in substantial injuries. However, the types and consequences of these injuries have not been systematically described.

**Objective:**

To describe the injury characteristics, outcomes, and health care burden associated with nonfatal injuries sustained during CPMSs and to better understand the consequences to patients, hospitals, and society at large.

**Design, Setting, and Participants:**

This retrospective case series of nonfatal injuries from 13 consecutive CPMSs (defined as ≥10 injured individuals) from 31 hospitals in the US from July 20, 2012, to August 31, 2019, used data from trauma logs and medical records to capture injuries, procedures, lengths of stay, functional impairment, disposition, and charges. A total of 403 individuals treated in hospitals within 24 hours of the CPMSs were included in the analysis. Data were analyzed from October 27 to December 5, 2021.

**Exposures:**

Nonfatal injuries sustained during CPMSs.

**Main Outcomes and Measures:**

Injuries and diagnoses, treating services, procedures, hospital care, and monetary charges.

**Results:**

Among the 403 individuals included in the study, the median age was 33.0 (IQR, 24.5-48.0 [range, 1 to >89]) years, and 209 (51.9%) were women. Among the 386 patients with race and ethnicity data available, 13 (3.4%) were Asian; 44 (11.4%), Black or African American; 59 (15.3), Hispanic/Latinx; and 270 (69.9%), White. Injuries included 252 gunshot wounds (62.5%) and 112 other injuries (27.8%), and 39 patients (9.7%) had no physical injuries. One hundred seventy-eight individuals (53.1%) arrived by ambulance. Of 494 body regions injured (mean [SD], 1.35 [0.68] per patient), most common included an extremity (282 [57.1%]), abdomen and/or pelvis (66 [13.4%]), head and/or neck (65 [13.2%]), and chest (50 [10.1%]). Overall, 147 individuals (36.5%) were admitted to a hospital, 95 (23.6%) underwent 1 surgical procedure, and 42 (10.4%) underwent multiple procedures (1.82 per patient). Among the 252 patients with gunshot wounds, the most common initial procedures were general and trauma surgery (41 [16.3%]) and orthopedic surgery (36 [14.3%]). In the emergency department, 148 of 364 injured individuals (40.7%) had 199 procedures (1.34 per patient). Median hospital length of stay was 4.0 (IQR, 2.0-7.5) days; for 50 patients in the intensive care unit, 3.0 (IQR, 2.0-8.0) days (13.7% of injuries and 34.0% of admissions). Among 364 injured patients, 160 (44.0%) had functional disability at discharge, with 19 (13.3%) sent to long-term care. The mean (SD) charges per patient were $64 976 ($160 083).

**Conclusions and Relevance:**

Civilian public mass shootings cause substantial morbidity. For every death, 5.8 individuals are injured. These results suggest that including nonfatal injuries in the overall burden of CPMSs may help inform public policy to prevent and mitigate the harm caused by such events.

## Key Points

**Question:**

What are the injury characteristics, outcomes, and health care services use among patients who have sustained nonfatal injuries in civilian public mass shootings?

**Findings:**

In this case series of 403 patients who sustained nonfatal injuries in 13 consecutive mass shootings (defined as ≥10 individuals injured) from 31 hospitals in the US (2012-2019), 252 patients (62.5%) had firearm injuries, 147 (36.5%) were admitted to a hospital, 148 (40.7%) underwent emergency department procedures, 95 (23.6%) underwent 1 surgical procedure, and 42 (10.4%) underwent multiple surgical procedures.

**Meaning:**

These findings suggest that the overall burden of mass shootings should not be limited to the number of deaths but should also incorporate nonfatal injuries, including those due to firearms and other trauma.

## Teaser

This case series evaluates the injury characteristics, outcomes, and resource use among individuals who have sustained nonfatal injuries in civilian public mass shootings.

## Introduction

Civilian public mass shootings (CPMSs) punctuate the firearm violence epidemic in the US and cause substantial numbers of deaths and injuries. Such shootings are the most common mass casualty events in the US,^1^ and they are rising in frequency,^2^ more than tripling from 2010 to 2019 compared with the previous decade.^3^ In 2021, there were nearly 700 CPMSs,^4^ and they continue to increase despite the COVID-19 pandemic.^5^ Mass shootings are a global problem, but the US claims 38% of the world’s 50 most deadly mass shootings^1^ and 31% of global perpetrators.^6^

Nonfatal gunshot wounds (GSWs) account for most firearm injuries in the US,^7^ yet most firearm violence studies focus on deaths,^8^ including those about mass shootings.^9,10,11,12^ Every day, more than 230 people sustain a nonfatal GSW in the US, or 1 every 7 minutes.^8^ The clinical importance of nonfatal GSW injuries by assault is amplified because most fatalities (61.2%) are suicides and most deaths (76.6%) occur outside the hospital.^7^ For every firearm-related fatality of all types in the US (not just those from CPMSs), 2.5 injured individuals are treated for nonlethal GSWs.^7^ Historically, for mass shootings alone, 1.5 to 1.6 patients sustain nonfatal GSWs for every death.^3,13^

To our knowledge, no study has comprehensively described nonfatal injuries (GSWs and non-GSWs) in CPMSs across multiple sites, and no study has described non-GSW injuries for individuals injured in CPMSs. Previous studies^14,15^ have reported only injuries from trauma registries, without an accounting of nontrauma activations, patients treated and released from the emergency department (ED), and noninjured patients from CPMSs. We herein report the injury characteristics, outcomes, and resource use of individuals who survive CPMS.

## Methods

In this case series, we identified 21 CPMSs with injuries from July 20, 2012, to August 31, 2019—15 from public databases and 6 from media and site investigators—based on the Congressional Research Service criteria (public setting, civilians injured indiscriminately, motive not for criminal or other gain).^16^ Although there have been more CPMSs in this period with fewer injuries, we focused on these because they had 10 or more injuries per shooting (treated at 53 receiving hospitals). We contacted local physicians in trauma and emergency medicine to participate. Data were available for 13 of 21 shootings (61.9%) and 31 of 53 primary recipient hospitals (58.5%). Sites, injured individuals, and exclusions are listed in the Figure. This study was classified as nonhuman participant research and did not require institutional review board (IRB) approval at the central hub (University of California, Irvine). Each data site obtained IRB approval. Individual patient consent was not required or obtained owing to use of deidentified data. This study followed the reporting guideline for case series.

**Figure.**
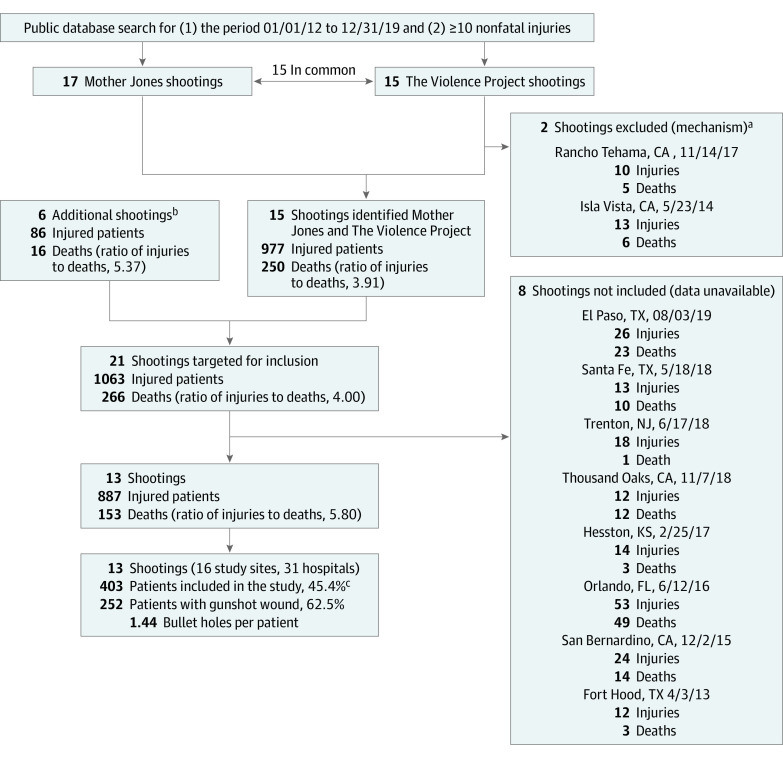
Study Flowchart of Sites and Patients Involved in Civilian Public Mass Shootings in the US, 2012-2019 ^a^Excluded given atypical mechanisms of mass murder (eg, stabbing, running down individuals in vehicle) and because some individuals were not chosen indiscriminately. ^b^Identified via study site coordinators, lay press sources, and public databases. ^c^Includes 252 (62.5%) with gunshot wounds, 112 (27.8%) with other trauma, and 39 (9.7%) without physical injury. Participants were eligible if they presented to a study site hospital within 24 hours of the mass shooting and did not die in the emergency department or during initial surgery if applicable. Perpetrators were excluded.

From the public database–identified CPMS, we identified nearby hospitals that were likely to have received injured individuals. The senior authors (M.P.C., C.K.K., and M.I.L.) then contacted ED or trauma directors from their professional networks, and they identified injured individuals they received and additional local hospitals that likely received them. We then contacted champions at those hospitals to find additional injured individuals. Investigators retrieved data from their electronic medical record system and trauma registry (if available) into REDCap, version 11.2.4 (Vanderbilt University). Injured individuals were included within 24 hours of the day and time of the incident. Those who died in the ED or during any initial surgical procedure and perpetrators were excluded. Investigators identified eligible participants via their trauma registry (if available) or billing department. Site investigators (P.O., D.R.M., D.S., T.H.G., C.H., B.P., M.L.W., U.A., A.Y., N.M.K., K.R.W., A.N.P., A.W.K., A.T., B.L., B.Y., and M.I.L.) determined which patients, both temporally and by type of injury, were injured in the CPMS. We analyzed deidentified data with descriptive statistics at the central site.

We collected 76 data elements in the following categories: demographics, insurance, initial site of care and trauma center status, mode of arrival, Emergency Severity Index (ESI) triage level at the initial ED, GSWs vs other injuries, number of penetrating wounds, organ systems injured, fractures (open and/or closed), Injury Severity Score (ISS), ED procedures and disposition, admission level of care, timing and number of surgical procedures, overall and intensive care unit length of stay, medical and surgical services involved in care, disposition after admission, *International Classification of Diseases, Ninth Revision,* and *International Statistical Classification of Diseases and Related Health Problems, Tenth Revision* codes, discharge diagnoses, index hospitalization charges, functional and cognitive disability, 30-day and 1-year readmissions, and elements to calculate the Charlson Comorbidity Index.^17^ Combined race and ethnicity were defined and collected by investigators to describe the demographics of injured individuals.^18^ This was deemed relevant to better report on demographics and compare (descriptively) with the racial and ethnic demographics of greater gun violence epidemic, which disproportionately affects Black and Latino individuals. Gun violence is a socialized issue. Investigators had the option of recording nonbinary gender, but none did; therefore, we report the biological sex of patients.

We used best-practice medical record abstraction methods.^19^ We trained abstractors, made specific definitions of cases, defined all variables, used standardized abstract fields with specific definitions, used continuous sampling of all eligible patients, had strategies to deal with missing or conflicting data, and obtained IRB approval at each site. We did not test interrater reliability because core investigators could not remotely access the data at the multiple sites to protect privacy. The abstractors were not blinded to the purpose of the study, but because there was no comparison of groups, there would be no potential for bias in data recording.

Data collection yielded 83% to 100% complete data for all variables except ESI, missing for 171 of 403 (42.4%); ISS, missing for 211 of 364 (58.0%); and charges, missing for 223 of 403 (55.3%). Because triage category was thought important for disaster planning, we extrapolated the missing ESI for the 171 patients based on diagnoses, admission data, services used, surgical procedures, ED procedures, and ISS.^20^ Without initial vital signs, we assigned patients an ESI of 3 rather than 2 (lower acuity) when uncertain. Individuals with GSWs were assumed to have an ESI of at least 3, given the high-risk mechanism, potential for consultations, and high likelihood of laboratory testing and intravenous fluid resuscitation.

We collected hospital charges for the index hospitalization where available, as a surrogate for cost. We estimated cost from published ratios of costs to charges for US hospitals^21^ and additional professional fees using published ratio estimates.^22^ We converted costs to 2021 US dollars using inflation rates.^23^

To report disabilities at discharge, investigators reviewed discharge summaries, operative notes, and diagnoses to record functional limitation(s) in use of the hand or arm, walking, cognition, or breathing as specified by a priori explicit definitions given to site investigators on how to determine if patients suffered from each of these disabilities. In addition, we assigned functional disability if patients had arm or leg fractures, because these would be splinted, even if not reported. We assumed cognitive impairment for patients diagnosed with traumatic brain injury. Breathing impairment on discharge was assumed for diagnoses of lung contusion, diaphragm injury, multiple rib fractures, hemothorax, pneumothorax, acute respiratory failure, or acute pulmonary embolism.

### Statistical Analysis

On completion of data entry, the deidentified data were aggregated and converted to Excel, version 15.45 (Microsoft Corporation), for analysis. This aggregation step precluded identification of individuals and ensured patient privacy.

## Results

We included 403 patients from 13 CPMS (Table 1). The median age was 33.0 (IQR, 24.5-48.0) years, with an age range of 1 to older than 89 years; 24 patients (6.0%) were younger than 18 years. A total of 209 patients (51.9%) were female and 194 (48.1%) were male. Among the 386 patients with race and ethnicity data available, 13 (3.4%) were Asian, 44 (11.4%) were Black or African American, 59 (15.3%) were Hispanic/Latinx, and 270 (69.9%) were White. Most patients (252 of 392 [64.6%]) were publicly insured or uninsured (data were missing for 11 patients). Comorbidities for this mostly young, healthy population yielded a mean (SD) Charlson Comorbidity Index of 0.6 (1.3), indicating a 97% estimated 10-year survival. The median Charlson Comorbidity Index was 0 (IQR, 0-1), indicating that 50% to 75% of patients had no reported comorbidities. Among the 403 patients included in the analysis, 252 (62.5%) had firearm injuries, 147 (36.5%) were admitted to a hospital, 169 (41.9%) underwent ED procedures, 95 (23.6%) underwent 1 surgical procedure, and 42 (10.4%) underwent multiple surgical procedures.

**Table 1.** Study Site Characteristics by Mass Shooting, Including Reported Number of Nonfatal Injuries, Deaths, Patients Included, and Initial Recipient Hospitals

Mass shooting	Date	Location	No. of reported injuries^a^	No. of reported deaths^a^	Ratio of injuries to deaths	No. (%) of patients included	Ratio of patients to deaths	No. of reported hospitals^a^	No. (%) of hospitals included
Route 91 Harvest festival	10/01/2017	Las Vegas, NV	596	58	10.3	219 (36.7)	3.76	12	8 (66.7)
Aurora Theater	07/20/2012	Aurora, CO	56	12	4.67	6 (10.7)	0.5	6	1 (16.7)
Fort Lauderdale Airport	01/06/2017	Fort Lauderdale, FL	46	5	9.2	46 (100)	9.2	1	1 (100)
Dayton	08/04/ 2019	Dayton, OH	38	9	4.22	38 (100)	4.22	7	7 (100)
Gilroy Garlic Festival	07/28/ 2019	Gilroy, CA	31	3	10.3	29 (93.5)	9.67	4	2 (50.0)
Power Ultra Lounge	07/01/2017	Little Rock, AR	26	0	NA^b^	11 (42.3)	NA^b^	4	1 (25.0)
Midland-Odessa	08/31/ 2019	Midland and Odessa, TX	24	7	3.43	13 (54.2)	1.86	4	1 (25.0)
First Baptist Church	11/05/ 2017	Sutherland Springs, TX	19	26	0.73	8 (42.1)	0.31	2	1 (50.0)
Marshall County High School	01/18/ 2018	Benton, KY	16	2	8.0	4 (25.0)	2.0	4	2 (50.0)
Marjory Stoneman Douglas High School	02/14/2018	Parkland, FL	13	17	0.76	13 (100)	0.76	3	3 (100)
Jacksonville Landing	08/26/2018	Jacksonville, FL	9	2	4.5	5 (55.5)	2.50	2	1 (50.0)
Tree of Life synagogue	10/27/ 2018	Pittsburgh, PA	7	11	0.64	7 (100)	0.64	2	2 (100)
Burnette Chapel	09/24/2017	Antioch, TN	6	1	6.0	4 (66.7)	4.0	2	1 (50.0)
Total	NA	NA	887	153	5.8	403 (45.4)	2.63	53	31 (58.5)

Abbreviation: NA, not applicable.

^a^ Includes reported nonfatal injuries, reported deaths (excluding perpetrators and those who died in the emergency department when known), and reported initial recipient hospitals that represent the authors’ appraisal of information provided by public databases (Mother Jones and The Violence Project), lay press sources, and study site coordinators.

^b^ A ratio cannot have a denominator of zero.

As shown in Table 1, there were 153 deaths associated with these 13 CPMS. According to public databases, a total of 887 individuals had nonfatal injuries, giving a ratio of injuries to deaths of 5.8. Given that we included 403 real patients (45.4%) who had documented medical care within 24 hours of each incident, we report 2.6 injured patients for every death.

Overall, 364 patients (90.3%) sustained an injury, of which 252 (69.2%) were GSWs and 112 (30.8%) were non-GSW injuries. Three patients had both types of injures, and we considered them to have GSWs. Thirty-nine patients (9.7%) did not sustain physical trauma. The mean (SD) of penetrating wounds per patient was 1.44 (0.82). The most frequent non-GSW mechanism of trauma was falling, stampeding, or trampling (69 [56.1%]), followed by nonpenetrating external trauma (39 [31.7%]), blunt force trauma (11 [8.9%]), and myocardial infarction (4 [3.3%]). The 494 body regions injured (mean [SD] of 1.35 [0.68] per patient) included extremities (282 [57.1%]), abdomen and/or pelvis (66 [13.4%]), head and/or neck (65 [13.2%]), chest (50 [10.1%]), and back (31 [6.3%]). There were 369 organ systems injured (mean [SD], 1.65 [1.07] per patient) among patients with GSWs (eTable 1 in the Supplement).

We collected ISS data from 153 of 364 injured patients (42.0%), exclusively from trauma registries, when available. Of these, 97 (63.4%) were GSWs, and 56 (36.6%) were non-GSW trauma. The median ISS for patients with GSWs was 9 (IQR, 4-14) vs 1 (IQR, 1-4) for patients with non-GSW injuries. Twenty-five patients (16.3%) had an ISS greater than 15. The ISS values ranged from 1 to 48 for patients with GSWs and 0 to 19 for those with non-GSW injuries (Table 2).

**Table 2.** Injury Characteristics of 364 Patients^a^

Injury type	No./total No. (%) of patients	No. of injuries (No. per patient)
Musculoskeletal trauma by GSW		
Total	97/252 (38.5)	177 (1.82)
Any fracture	83/252 (32.9)	163 (1.96)
Open fracture^b^	58/83 (63.0)	94 (1.62)
Tendon laceration	12/252 (4.8)	12 (1.00)
Finger amputation	2/252 (0.8)	2 (1.00)
Neurological trauma by GSW		
Total	29/252 (11.5)	36 (1.24)
Peripheral nerve injury	22/252 (8.7)	22 (1.00)
Intracranial traumatic brain injury	6/252 (2.4)	13 (2.17)^c^
Spinal cord injury	1/252 (0.4)	1 (1.00)
Musculoskeletal/neurological trauma by non-GSW injury		
Total	28/112 (25.0)	30 (1.07)
Fracture	9/112 (8.0)	11 (1.22)
Dislocation	5/112 (4.5)	5 (1.00)
Closed head injury or concussion	14/112 (12.5)	14 (1.00)

Abbreviation: GSW, gunshot wound.

^a^ Includes 252 with GSWs and 112 with only nonballistic trauma (fall, stampede, trampling, blunt, or external). Injury Severity Scores (ISS) are calculated as the sum of squares of the 3 most injured Abbreviated Injury Score body regions. Major trauma (or multiple trauma) is defined as ISS greater than 15. A total of 153 patients with ISS available had major trauma, including 24 of 97 (24.7%) with GSWs and 1 of 56 (1.8%) with non-GSW injuries. Median ISS for all GSW injuries was 4 (IQR, 1-9 [range, 1-48]); for all non-GSW injuries, 1 (IQR, 1-4 [range, 0-19]).

^b^ Fifty-eight of 81 patients with GSWs and any fracture (71.6%) had open fractures. There were no recorded open fractures in patients with non-GSW injuries. Given that open fracture is a subtype of total fractures, these were counted only once in the calculation of total musculoskeletal trauma.

^c^ Includes 13 intracranial (epidural, subdural, subarachnoid, intraparenchymal, and cerebral hemorrhage/laceration) injuries by GSWs.

Approximately half of the patients presented to trauma centers (209 [51.9%])—182 of whom (87.1%) presented initially to level I trauma centers—with the remainder (194 [48.1%]) treated at community (nontrauma) hospitals. Of those initially treated at community hospitals, 10 of 183 adults (5.5%) and 4 of 11 children (36.4%) required transport to a level I trauma center. One hundred seventy-eight patients arrived by ambulance (53.1%), although almost as many arrived by private vehicle or walked in (146 [43.6%]). Sixty-one patients (15.1%) were triaged as ESI level 1 (life-threatening injuries needing immediate resuscitation to prevent death^20^); 71 (17.6%), ESI level 2 (high risk or altered mental status or severe pain and/or distress^20^); 149 (37.0%), ESI level 3 (medium acuity); 85 (21.1%), ESI level 4 (low acuity); and 37 (9.2%), ESI level 5 (low acuity).

Among the 364 injured patients, 148 (40.7%) had an ED procedure and underwent 199 procedures (mean [SD], 1.34 [0.72] per patient) (Table 3 and eTable 2 in the Supplement). Ninety-seven patients (26.6%) required casting and/or splinting, and 61 (16.7%) had at least 1 laceration repair (mean [SD], 1.23 [0.74] per patient). Fourteen patients (3.8%) had endotracheal intubation, 12 (3.3%) had tube thoracostomy, and 12 (3.3%) had central intravenous line placement in the ED. Overall, 147 individuals (36.5%) were admitted to a hospital, 95 (23.6%) underwent 1 surgical procedure, and 42 (10.4%) underwent multiple procedures (mean [SD], 1.82 [1.10] per patient). Nearly all patients who underwent surgery (93 of 95) had GSWs. Of all 252 patients with GSWs, 93 (36.9%) had surgery, whereas 2 of 112 with non-GSW injuries (1.8%) did. The first surgical procedure was performed within 24 hours for 89 of 95 patients (93.7%), and 42 of 95 (44.2%) had additional operations. A total of 173 surgical procedures were performed in the operating room (mean [SD], 1.82 [2.01] per patient; maximum of 14 in 1 patient). General or trauma surgery was the most common initial procedure (41 of 252 [16.3%]), followed by orthopedic surgery (36 of 252 [14.3%]), although this order was reversed for additional procedures. Vascular and hand surgery were third and fourth in frequency, respectively, for both first and additional surgical procedures. The largest increases in frequency from first to additional procedures were for plastic (6.1%) and urological (4.4%) surgery.

**Table 3.** Procedures in the ED and OR

Procedure type	Patients, No./total No. (%)	No. of procedures (No. per patient)
ED procedures for all injured patients		
Total^a^	148/364 (40.7)	199 (1.35)
Cast or splint	62/364 (17.0)	62 (1.00)
Laceration repair	61/364 (16.7)	75 (1.13)
Endotracheal intubation	14/364 (3.8)	14 (1.00)
Central intravenous line	12/364 (3.3)	12 (1.00)
Tube thoracostomy	12/364 (3.3)	12 (1.00)
Fracture/dislocation reduction	10/364 (2.7)	10 (1.00)
OR procedures for all injured patients		
Any	95/364 (26.1)	173 (1.82)
Additional surgery	42/95 (44.2)	58 (1.38)
First surgery within 24 h^b^	89/95 (93.7)	89 (1.00)
OR procedures for patients with GSWs		
Any	93/252 (36.9)	169 (1.81)
First	93/252 (36.9)	112 (1.20)
Additional	41/252 (16.3)	57 (1.39)
OR procedures for patients with non-GSW injuries		
Any	2/112 (1.8)	4 (2.00)
First	2/112 (1.8)	3 (1.50)
Additional	1/112 (0.9)	1 (1.00)

Abbreviations: ED, emergency department; GSW, gunshot wound; OR, operating room.

^a^ Fourteen ED procedures not listed and contributing to the total were blood product transfusion (n = 4), procedural sedation (n = 4), foreign body removal (n = 4), and arterial line placement (n = 2).

^b^ Eighty-eight of 93 (94.6%) OR procedures for GSWs were performed within 24 hours vs 1 of 2 (50%) for individuals with non-GSW injuries.

Providing health care for all patients required 1350 clinical services (mean [SD], 3.35 [2.16] per patient). All patients received emergency care. In order of frequency, 303 patients (75.2%) received diagnostic radiology services; 135 (33.5%), general or trauma surgery; 115 (28.5%), internal or hospital medicine services; 95 (23.6%), anesthesiology services; 93 (23.1%), orthopedic surgery; 87 (21.6%), surgery from other specialties; and 50 (12.4%), critical care. Of the other surgical specialties, vascular was the most common (n = 25), followed by hand (n = 12), cardiothoracic (n = 11), neurological and/or spine (n = 11), plastic (n = 9), otolaryngology–head and neck (n = 7), interventional radiology (n = 5), urology (n = 4), and ophthalmology (n = 3). Among general and trauma surgery consultations, 70 patients (51.9%) had surgery, of whom 43 (31.9%) had general and trauma procedures and 27 (20.0%) had procedures from another specialty. Among orthopedic consultations, 43 (46.2%) had orthopedic surgery, 11 (11.8%) had other surgery, and 10 (10.7%) had closed fracture and/or dislocation reduction in the ED. Thirty patients (7.4%) received psychiatry, psychology, and/or ethics consultations; 17 (4.2%), consultations from a medical specialty; 11 (2.7%), pediatric consultations; 8 (2.0%), neurological consultations; and 3 (0.7%), obstetrics and gynecology consultations.

Table 4 shows hospital dispositions, lengths of stay in the hospital and intensive care unit, and index hospitalization charges. Of the 364 injured patients, 147 (40.4%) were admitted (or transferred for admission). Of these, 8 (5.4%) had non-GSW injuries, and 1 (0.7%) had a myocardial infarction and was admitted without injury. There were no inpatient deaths after admission. For 143 admitted patients, 16 (11.2%) were readmitted or had a subsequent ambulatory procedure within 30 days at the same hospital. Within 1 year, 66 (46.2%), patients were readmitted to the same hospital. Median hospital length of stay was 4.0 (IQR, 2.0-7.5) days; for 50 patients in the intensive care unit, 3.0 (IQR, 2.0-8.0) days (13.7% of injuries and 34.0% of admissions). One hundred sixty patients (44.0%) had functional disability at discharge, and 19 (13.3%) were referred to long-term care.

**Table 4.** Disposition From ED and Hospital, Admission Level of Care, LOS Overall and in ICU, Charges, Disability at Discharge, and Readmissions

Variable	Patients, No. (%)
ED disposition, No./total No. (%) of patients	
Home	256/403 (63.5)
Admitted^a^	147/403 (36.5)
Initial admission level of care**,** No./total No. (%) of patients^b^	
Medical-surgical or general floor	78/143 (54.5)
ICU	44/143 (30.8)
Step down/intermediate	21/143 (14.7)
Any ICU admission, No./total No. (%) of patients^c^	50/143 (35.0)
LOS for initial admission, d^b^	
All admitted patients (n = 143)	
Total (range)	920 (1-67)
Mean (SD)	6.4 (8.4)
Median (IQR)	4.0 (2.0-7.5)
ICU stay (n = 50)	
Total (range)	333 (1-34)
Mean (SD)	6.7 (7.9)
Median (IQR)	3.0 (2.0-8.0)
Inpatient disposition, No./total No. (%) of patients^b^	
Home	118/143 (82.5)
Rehabilitation or skilled nursing facility	19/143 (13.3)
Transfer to another acute care hospital	6/143 (4.2)
Functional disability at discharge, No./total No. (%) of patients	
Total	160/364 (44.0)
Ambulation or lower extremity	91/364 (25.0)
Hand and/or arm use	64/364 (17.6)
Breathing	31/364 (8.5)
Cognitive	12/354 (3.3)
Gastrointestinal (ostomies, dysphagia)	4/364 (1.1)
No. of disabilities (No. per patient)^b^	
Total	202 (1.26)
Ambulation or lower extremity	91 (1.00)
Hand and/or arm use	64 (1.00)
Breathing	31 (1.00)
Cognitive	12 (1.00)
Gastrointestinal (ostomies, dysphagia)	4 (1.00)
Readmission status, No./total No. (%) of patients^b^	
30-d	16/143 (11.2)
1-y	66/143 (46.2)
No. of readmissions (No. per patient)^b^	
30-d	16 (1.00)
1-y	79 (1.20)
Index hospitalization charges, $^d^	
Total (range)	11 695 715 (570-1 277 983)
Mean (SD)	64 976 (160 083)
Median (IQR)	9311 (2496-40 907)

Abbreviations: ED, emergency department; ICU, intensive care unit; LOS, length of stay.

^a^ Nearly all admissions were for gunshot wounds (GSWs): 138 of 147 (93.9%) vs 8 of 112 (7.1%) for non-GSW injures and 1 of 39 (2.6%) for those without traumatic injury. The admission results presented herein are aggregated.

^b^ Unable to report status for 4 transfer admissions.

^c^ Six patients required ICU stay despite being initially admitted to a different level of care. A total of 50 of 143 (35.0%) admitted patients used the ICU.

^d^ Charges for initial hospitalization in 2020 US dollars. Data available for 180 of 403 patients (44.7%).

We report substantial hospital charges (mean [SD], $64 976 [$160 083] per patient) from shootings spanning a decade. To estimate costs, we first added professional fees (which we did not collect, estimated at 32% of hospital charges),^22^ then reduced charges by a factor of 3.4 to estimate costs.^21^ Finally, we adjusted for inflation of 26.4% (2012-2021 US Bureau of Labor statistics).^23^ Therefore, our mean (SD) per-patient reported charge for index hospitalization of $64 976 equates to an estimated $31 885 in 2021 costs for facility and professional components. This does not include costs of readmissions. Further, 9 patients (13.3%) required subsequent nonacute care, adding to the financial burden.

## Discussion

Three other studies^14,15,24^ have reported injuries from CPMSs in the US. In this largest case series to date, we report the injuries, ED and hospital resources used, and outcomes of 403 patients injured in 13 mass shootings in the US from 2012 to 2019. This study highlights the high ratio of injuries to deaths common to CPMSs, along with the burden of injury and associated use of health care services. These estimates are higher than the previously reported ratios of 1.5 to 1.6 for CPMSs^3,13^ and similar to reported ratios of 2.5 for all firearm violence and 2.8 for firearm assault.^7^ Among the 403 cases in our study, 2.60 patients received medical treatment for every reported CPMS fatality. However, the total number of injured individuals recorded by public databases was 887 (with the same 153 deaths), yielding a ratio of as much as 5.80 (Table 1).

Trauma registry data do not provide a comprehensive picture of all individuals affected by CPMSs. Sarani et al^14^ described patients in a trauma center registry for individuals involved in CPMSs from 1999 to 2017, including 31 events with 191 patients, but reported only trauma activations and excluded graze GSWs and nonballistic trauma. Similarly, Knickerbocker et al^15^ reported wound patterns and resource use for 2 of the CPMSs included in the present study, with 19 injured individuals from trauma centers. Not surprisingly, they found higher rates of fatality (55%) and ICU admission (32%) in these 2 events. To understand the true burden of CPMSs, we examined trauma centers and the nearly 50% of patients who presented to nontrauma hospitals. In addition, capturing ED, hospital, and surgical procedures and charges gives a more complete assessment of CPMS to better inform ED and hospital preparedness.

We could not obtain data from the Pulse Nightclub shooting in Orlando, Florida, but Smith et al^24^ reported comparisons with this more lethal shooting. Of 102 total injured individuals, there were 49 deaths (48%) compared with 14.7% at our sites, and only 19 patients from the Orlando shooting (18.6%) were treated and released from the ED (vs 62.5% in the present study). Although we included all patients from mass shootings, both injured and not, Smith et al^24^ focused on GSWs only. Whereas 82.4% of their admitted patients needed surgery in the first 24 hours, the corresponding percentage in our study was 64.6%.

Many individuals involved in CPMSs arrive at the nearest hospital by non–emergency medical services transport (46.3%).^25^ Among the 403 patients described herein, 209 (51.9%) presented to trauma centers—182 of whom (87.1%) presented initially to level I trauma centers—and the remainder (194 [48.1%]) presented to community hospitals. However, many patients who were injured in the Route 91 Harvest music festival shooting in Las Vegas, Nevada, went to level II trauma centers, where we were administratively denied access. We documented only 10 of 183 adults (5.5%) and 4 of 11 children (36.4%) who were transferred from community hospitals to trauma centers, but this is likely an underestimate. All types of EDs should prepare for a CPMS-related influx of ambulance and walk-in patients, particularly those closest to an incident.^25^

Our mostly non-Hispanic White proportion of injured patients (69.9%) from CPMSs contrasts with the largely Black and Hispanic or Latino (64%) patients among all nonfatal firearm injuries.^26^ The proportion of publicly insured or uninsured patients in our study (64.3%) was the same as that for all firearm injuries.^27^ Our distribution of CPMS ESI triage categories reflects substantially higher acuity than a US population treated in the ED for nondisaster incidents.^28^ Previous work on all US shootings has shown similar proportions of ISS of greater than 15 as our data (14.3% vs 16.3% herein), validating the severity of injury for GSWs.^7^ The difference in median ISS values for GSWs vs non-GSWs found herein reflects the intuitive conclusion that patients with GSWs were more severely injured, but 1 individual with a trampling injury had a high ISS of 19.

Beyond the index admission, 46.2% of patients were readmitted within 1 year (1.2 per patient) and 11.2% were readmitted within 30 days, rates that are higher than those of previous reports (7.6% for all nonfatal firearm injuries in the US).^28^ Previous work^29^ has documented a 90-day acute care readmission rate of 20%—which, as expected, is intermediate between our 30-day and 1-year results. In the previous study on readmissions alone,^29^ there was a substantially lower cost per case compared with the present study ($9357 vs $64 976, respectively), as well as a shorter length of hospital stay (4.48 vs 6.4 days, respectively). Cook et al^30^ reported rising hospital charges for index hospitalizations for patients with GSWs from $30 000 in 2004 to $56 000 in 2013. Specific comparisons are difficult because converting any hospital charges to the method used herein for costs is likely different. However, costs and charges likely remain substantial and may be rising.

### Limitations

This study has some limitations. We were unable to collect data from 8 CPMSs owing to lack of hospital research infrastructure, lack of staff owing to the COVID-19 pandemic, refusal of some health systems to allow physicians to participate in the project for public relations reasons, age of some medical records, and legacy electronic medical record systems. Without a list of the destination hospitals for individuals involved in CPMSs, our reliance on personal networks to identify recipient hospitals may mean that we missed data on patients at some community hospitals. Some patients, especially in Las Vegas, were not registered because electronic medical record systems during disasters often cannot keep up with volume and pace.^31,32^ We were unable to validate data entry or to describe κ values owing to local IRB and privacy restrictions.

Hospital charges were unavailable for 55.3% of patients owing to changes in financial systems for older shootings. The ISS values were unavailable for 54.6% of patients because only hospitals with trauma registries could report this. We did not have access to long-term disability status for injured patients. We were unable to track patients’ readmissions (if any) to other hospitals beyond the initial sites of index hospitalizations. We also did not gather data on the reasons for readmissions or subsequent surgical procedures or services involved. However, comments indicate that some of these were repeated orthopedic procedures, as would be expected. Our extrapolations of physical and cognitive disabilities from reported diagnoses could be challenged, although site investigators specifically reported patients with disabilities at discharge.

We did not gather data from CPMSs that had fewer than 10 injured individuals during this period, because this would have made IRB approval and data collection infeasible. Our report may not reflect the consequences among all individuals injured in CPMSs. Given the methodological limitations of a case series, future observational studies permitting statistical comparisons would be useful for further investigation of the injury characteristics and outcomes among individuals who survive CPMSs.

## Conclusions

Mass shootings in the US cause enormous burden to patients, EDs, hospitals, and society at large. Nearly 6-fold more individuals are injured than those who die. Although two-thirds sustain GSWs, one-third have other injuries. One-third require admission to a hospital and almost half are readmitted. More than one-third of patients with GSWs undergo surgery, and almost one-half have a disability at discharge. Hospital charges are substantial. These results can inform preparation and responses for prehospital, ED, and hospital care. Given the limitations of our data collection, we recommend establishing a national data registry that addresses the consequences of mass shootings.

## Notes

Supplement. **eTable 1.** Body Regions and Organ Systems Injured **eTable 2.** First and Additional Surgical Procedures in Operating Room for Patients With GSWs
