# Supplementary material for: Injury Characteristics, Outcomes, and Health Care Services Use Associated With Nonfatal Injuries Sustained in Mass Shootings in the US, 2012-2019
Source: JAMA Netw Open. 2022 May 27;5(5):e2213737. doi: 10.1001/jamanetworkopen.2022.13737 (PMC9142871; doi:10.1001/jamanetworkopen.2022.13737)
Supplement: Supplement. — eTable 1. Body Regions and Organ Systems Injured eTable 2. First and Additional Surgical Procedures in Operating Room for Patients With GSWs [file jamanetwopen-e2213737-s001.pdf]

Supplementary Online Content

Czaja MP, Kraus CK, Phyo S, et al. Injury characteristics, outcomes, and health care services use associated with nonfatal injuries sustained in mass shootings in the US, 2012-2019. *JAMA Netw Open*. 2022;5(5):e2213737.
doi:10.1001/jamanetworkopen.2022.13737

eTable 1. Body Regions and Organ Systems Injured
eTable 2. First and Additional Surgical Procedures in Operating Room for Patients With GSWs

This supplementary material has been provided by the authors to give readers additional information about their work.

© 2022 Czaja MP et al. *JAMA Network Open*.

eTable 1. Body Regions and Organ Systems Injured

**Body region or organ system injured**	**No./total No. (%) of patients with injuries**	**No./total No. (%) of body regions or organ systems injured**
Body regions injured by GSW and non-GSW injuries (1.36 per patient)		
Extremity	282/364 (77.5)	282/494 (57.1)
Abdomen and/or pelvis	66/364 (18.1)	66/494 (13.4)
Head, face, and/or neck	65/364 (17.9)	65/494 (13.1)
Chest	50/364 (13.7)	50/494 (10.1)
Back	31/364 (8.5)	31/494 (6.3)
Organ systems injured by GSW (1.46 per patient)		
Muscle	147/252 (58.3)	147/369 (39.8)
Bone	83/252 (32.9)	83/369 (22.5)
Peripheral vascular	34/252 (13.5)	34/369 (9.2)
Neurologic	29/252 (11.5)	29/369 (7.9)
Lung, trachea, and/or bronchi	21/252 (8.3)	21/369 (5.7)
Bowel, stomach, esophagus, and/or pancreas	18/252 (7.1)	18/369 (4.9)
Face and/or otorhinolaryngologic	13/252 (5.1)	13/369 (3.5)
Genital and/or genitourinary	6/252 (2.4)	6/369 (1.6)
Liver and/or biliary	6/252 (2.4)	6.369 (1.6)
Diaphragm	5/252 (2.0)	5/369 (1.3)
Kidney and/or adrenal	3/252 (1.2)	3/369 (0.8)
Spleen	3/252 (1.2)	3/369 (0.8)
Heart and/or great vessels	1/252 (0.4)	1/369 (0.3)

Abbreviation: GSW, gunshot wound.

© 2022 Czaja MP et al. *JAMA Network Open*.

eTable 2. First and Additional Surgical Procedures in Operating Room for Patients With GSWs[a]

Procedure	No./total/No. (%) of patients	No./total No. (%) of procedures	Change from first procedure, %
First OR procedure			
General and trauma	41/252 (16.3)	41/112 (36.6)	NA
Orthopedic	36/252 (14.3)	36/112 (32.1)	NA
Vascular	14/252 (5.5)	14/112 (12.5)	NA
Hand	8/252 (3.2)	8/112 (7.1)	NA
Cardiothoracic	7/252 (2.8)	7/112 (6.3)	NA
Neuro/spine	2/252 (0.8)	2/112 (1.8)	NA
Other[b]	2/252 (0.8)	2/112 (1.8)	NA
Plastic	1/252 (0.4)	1/112 (0.9)	NA
Urological	1/252 (0.4)	1/112 (0.9)	NA
Additional OR procedure			
Orthopedic	17/93 (18.3)	17/57 (29.8)	−2.3
General/trauma	14/93 (15.0)	14/57 (24.6)	−12.0
Vascular	8/93 (8.6)	8/57 (14.0)	+1.5
Hand	5/93 (5.4)	5/57 (8.8)	+1.7
Plastic	4/93 (4.3)	4/57 (7.0)	+6.1
Cardiothoracic	3/93 (3.2)	3/57 (5.3)	−0.8
Urological	3/93 (3.2)	3/57 (5.3)	+4.4
Other[b]	3/93 (3.2)	3/57 (5.3)	+3.5
Neurological and/or spine	0	0	−1.8

Abbreviations: GSW, gunshot wound; OR, operating room.

[a]Excludes 2 patients with non-GSW injury, accounting for 3 initial surgical procedures (2 orthopedic and 1 general or trauma) and 1 additional orthopedic procedure. For all 95 patients undergoing any surgical procedure in the OR, 173 discrete trips to the OR occurred for patients with and without GSWs, regardless of how many types of procedures were performed. Patients with GSWs accounted for 170 OR trips vs 3 for patients with non-GSW injuries. Median number of trips was 1 (IQR, 102); mean (SD), 1.82 (2.00); and range, 1 to 14.

[b]Includes maxillofacial, otolaryngology, head and neck, and ophthalmology.

© 2022 Czaja MP et al. *JAMA Network Open*.
